# NSD3S stabilizes MYC through hindering its interaction with FBXW7

**DOI:** 10.1093/jmcb/mjz098

**Published:** 2019-10-22

**Authors:** Valentina Gonzalez-Pecchi, Albert K Kwan, Sean Doyle, Andrey A Ivanov, Yuhong Du, Haian Fu

**Affiliations:** 1 Graduate Program in Cancer Biology, Emory University, Atlanta, GA, USA; 2 Department of Pharmacology and Chemical Biology, Emory Chemical Biology Discovery Center, Emory University, Atlanta, GA, USA; 3 Winship Cancer Institute, Emory University, Atlanta, GA, USA; 4 Department of Hematology & Medical Oncology, Emory University, Atlanta, GA, USA

**Keywords:** cancer, FBXW7, MYC, NSD3S, protein–protein interaction

## Abstract

The MYC transcription factor plays a key role in cell growth control. Enhanced MYC protein stability has been found to promote tumorigenesis. Thus, understanding how MYC stability is controlled may have significant implications for revealing MYC-driven growth regulatory mechanisms in physiological and pathological processes. Our previous work identified the histone lysine methyltransferase nuclear receptor binding SET domain protein 3 (NSD3) as a MYC modulator. NSD3S, a noncatalytic isoform of NSD3 with oncogenic activity, appears to bind, stabilize, and activate the transcriptional activity of MYC. However, the mechanism by which NSD3S stabilizes MYC remains to be elucidated. To uncover the nature of the interaction and the underlying mechanism of MYC regulation by NSD3S, we characterized the binding interface between both proteins by narrowing the interface to a 15-amino acid region in NSD3S that is partially required for MYC regulation. Mechanistically, NSD3S binds to MYC and reduces the association of F-box and WD repeat domain containing 7 (FBXW7) with MYC, which results in suppression of FBXW7-mediated proteasomal degradation of MYC and an increase in MYC protein half-life. These results support a critical role for NSD3S in the regulation of MYC function and provide a novel mechanism for NSD3S oncogenic function through inhibition of FBXW7-mediated degradation of MYC.

## Introduction

*c-MYC* (MYC) encodes a transcription factor and was one of the first oncogenes to be discovered in human cancers (Vennstrom and Bishop, 1982; Land et al., 1983). MYC functions by altering cellular characteristics associated with oncogenic transformation, such as proliferation (Karn et al., 1989; Iritani and Eisenman, 1999), apoptosis (Evan et al., 1992), metabolism (Shim et al., 1997; Hu et al., 2011), and angiogenesis (Baudino et al., 2002). Dysregulation of MYC activity, which occurs most commonly via *MYC* gene amplification, is found in a variety of human cancer types: on average, 50% of human cancers have increased expression of MYC. High MYC expression levels are furthermore correlated with increased tumor aggressiveness (Spencer and Groudine, 1991; Vita and Henriksson, 2006).

The MYC protein is composed of four conserved regions known as MYC boxes (MBI, MBII, MBIII, and MBIV). The C-terminal portion of MYC contains a basic helix-loop-helix-leucine zipper domain (bHLH-LZ) that is responsible for heterodimerization with MYC-associated factor x (MAX) (Luscher and Larsson, 1999). The MYC/MAX complex binds to specific sequences in the DNA known as enhancer box (E-box) sequences, and recruits transcriptional co-activators to drive expression of MYC target genes (Blackwell et al., 1990; Amati et al., 1993; Eisenman, 2001).

MYC protein levels are tightly controlled by several mechanisms, including post-translational modifications (PTMs) and protein–protein interactions (PPIs). An example of such a PTM is phosphorylation of serine 62 (S62) by extracellular-regulated kinase 1 (ERK1) and ERK2, which leads to the stabilization of MYC (Sears et al., 2000). This phosphorylation event creates a consensus region for subsequent phosphorylation of threonine 58 (T58) by glycogen synthase kinase 3β (GSK3β) (Yeh et al., 2004), which marks MYC for degradation (Gregory et al., 2003). Ultimately, phosphorylation of T58 and dephosphorylation of S62 (Liu and Eisenman, 2012) provide a binding site for F-box and WD repeat domain containing 7 (FBXW7), a substrate recognition subunit of SCF E3 ubiquitin ligase complexes , which targets MYC for ubiquitin-mediated proteasomal degradation (Welcker et al., 2004; Yada et al., 2004). Through its intricate interactions with FBXW7 and other regulatory proteins, MYC serves as a central node that integrates upstream signaling events to control diverse intracellular transcriptional programs during normal physiological development. Dysregulation of MYC protein levels through MYC overexpression or reduced degradation may lead to multiple diseases, including cancer. Thus, understanding how the MYC protein stability is properly controlled through these molecular interactions has broad implications for the regulation of cell growth under physiological and pathological conditions. Our previous work on the establishment of the OncoPPi network revealed a new member of the MYC regulatory proteins, nuclear receptor binding SET domain protein 3 (NSD3) (Li et al., 2017).

NSD3 is a lysine methyltransferase that belongs to the family of NSD proteins, including NSD1, NSD2, and NSD3 (Stec et al., 2001). NSD3 is thought to act as an oncogene, as it is frequently amplified in breast, lung, and pancreatic cancers (Garcia et al., 2005; Tonon et al., 2005). NSD3 has three isoforms: NSD3 long (NSD3L) that encodes the full-length protein with histone methyltransferase catalytic activity, NSD3 short (NSD3S) that lacks the catalytic SET domain-containing C-terminal fragment of the protein, and a testis-specific isoform named Whistle (Angrand et al., 2001; Stec et al., 2001). Interestingly, unique functions for the NSD3S isoforms have been reported, including a role in oncogenesis that is independent of methyltransferase activity. In leukemia cells, NSD3S has been shown to be essential for cancer progression by bridging the interaction between the bromodomain containing protein 4 (BRD4) and chromodomain helicase DNA binding protein 8 (CHD8) (Shen et al., 2015). In nuclear protein in testis (NUT)-midline carcinoma, NSD3 has been found to generate an oncogenic fusion with NUT (NSD3-NUT), and the portion of NSD3 present in the fusion is almost the complete NSD3S sequence. The oncogenic fusion decreases cellular differentiation and increases proliferation of NUT-midline carcinoma cell lines (French et al., 2014; Suzuki et al., 2014). We previously found that the same noncatalytic isoform of NSD3, NSD3S, was able to interact with MYC and promote its protein stability (Li et al., 2017). However, the precise mechanism by which NSD3S regulates MYC stability remains unclear. To address this issue, we have defined the PPI interfaces between NSD3S and MYC and revealed major binding sites on MYC and NSD3S, respectively. Mechanistic studies showed that NSD3S competes with FBXW7 for binding to MYC, providing a potential model for the regulation of MYC stability through FBXW7 (Yeh et al., 2018). This functional connection between MYC and NSD3S provides a novel link between two oncogenic proteins that are known to drive cancer cell survival and may serve as a promising target for therapeutic intervention in MYC-driven tumors.

## Results

### NSD3S binds to MYC at a distinct site

**Figure 1:**
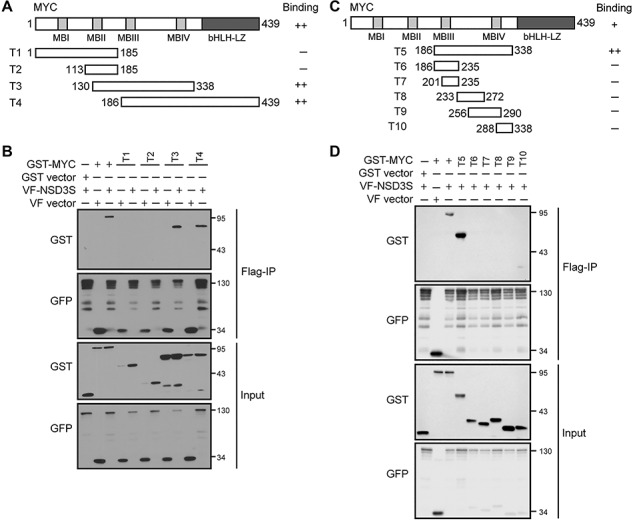
Identification of MYC sequence that binds to NSD3S. (**A**) Diagram of MYC protein domains showing truncations used in the study labelled with residue numbers. (**B**) Determination of MYC fragments involved in NSD3S binding. VF-NSD3S was co-expressed with GST-MYC and MYC truncations (T1–T4) in HEK293T cells. The VF-NSD3S protein complex was subjected to Flag-IP. The presence of GST-MYC proteins in the NSD3S complex was detected by western blotting with anti-GST antibody. Expression levels of VF-NSD3S and GST-MYC constructs were shown in the input. (**C**) Diagram of refined truncations of MYC (amino acids 186–338). (**D**) Determination of MYC fragments (T5–T10) for NSD3S binding. Flag-IP and western blotting were carried out as described in **B**.

To understand the molecular basis of the MYC/NSD3S interaction, we examined MYC structural elements that are required for NSD3 binding through deletion analyses. MYC contains four conserved domains with defined functions for MYC regulation. Four large truncations of MYC were first generated: T1 containing MBI and MBII (1–185), T2 with only MBII (113–185), T3 spanning MBII to MBIV (130–338), and T4 with MBIII, MBIV, and the basic helix loop helix domain (186–439) (Figure 1A). Co-expression of glutathione S-transferase (GST)-MYC truncations with Venus-Flag (VF)-tagged NSD3S was followed by immunoprecipitation with anti-Flag antibodies (Flag-IP) to pull down the NSD3S complex. Consistent with our previous report (Li et al., 2017), full-length MYC was confirmed to interact with NSD3S (Figure 1B), which served as a positive control for binding site mapping. Fragments T3 (130–338) and T4 (186–439) of MYC showed similar strength of interaction with NSD3S as the full-length MYC (Figure 1B). Conversely, MYC truncations lacking region T3 or T4 exhibited reduced binding to NSD3S (Figure 1B). These results suggest that the region shared between truncations T3 and T4, residues 186–338 of MYC, are required for binding to NSD3S (Figure 1A). To further narrow down the region of MYC that mediates the NSD3S/MYC interaction, we generated refined MYC fragments spanning residues 186–338 (Figure 1C) and performed further pulldown experiments. While full-length MYC and fragment 186–339 were found to interact with NSD3S, none of the smaller MYC truncations were detected in complex with NSD3S (Figure 1D). Together, these results suggest that the internal region, residues 186–338, between MBIII and MBIV of MYC is involved in the interaction with NSD3S.

### A 15-amino acid peptide of NSD3S mediates MYC binding

**Figure 2:**
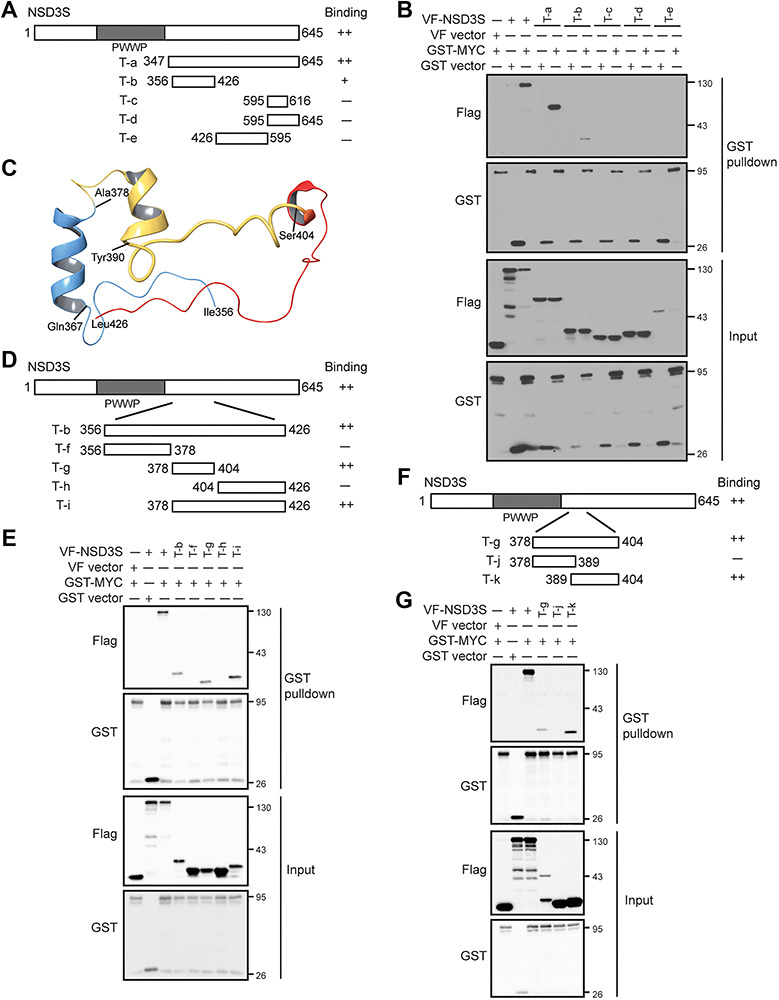
Determination of structural elements of NSD3S for MYC binding. (**A**) Diagram of NSD3S protein domains and truncations used for the study. Fragments (T-a to T-e) are labelled with residue numbers, and binding activity is indicated with plus signs. (**B**) Determination of NSD3S fragments responsible for MYC binding by GST pulldown in HEK293T cells. GST-MYC protein was isolated with glutathione beads, and binding of VF-NSD3S truncations to GST-MYC was detected using anti-Flag antibody. Binding of MYC to full-length NSD3S was included as a control. Expression of test proteins is shown as the input. (**C**) Computational model of NSD3S 356–426 with predicted structural elements as indicated: 356–378 (blue), 378–404 (yellow), and 404–426 (red). (**D**) Diagram of NSD3S truncations (T-f to T-i) of fragment T-b (356–426), which were designed according to the computational model. Truncations are labelled based on the residue number. (**E**) Characterization of the binding between VF-NSD3S and GST-MYC by GST pulldown in HEK293T cells. GST beads were used to pull down GST-MYC protein, and the binding to NSD3S truncations was determined by western blotting using anti-Flag antibody. Binding of MYC to full-length NSD3S and fragment T-b (356–426) was included as a control and protein expression was determined in the input. (**F**) Diagram of refined truncations spanning fragment T-g (378–404) of NSD3S. (**G**) Characterization of refined NSD3S peptides responsible for binding to MYC by the GST pulldown assay in HEK293T cells. GST-MYC was pulled down, and binding of VF-NSD3S peptides was detected by western blotting with anti-Flag antibody. Binding of MYC to full-length NSD3S and fragment T-g (378–404) was included as a control, and protein expression was determined in the input.

The C-terminal 347–645 amino acid region of NSD3S was previously found to mediate binding to MYC (Li et al., 2017). Further characterization of the residues on NSD3S involved in MYC binding may reveal the molecular basis for the interaction and identify strategies to manipulate the interaction for functional studies. We used a computational molecular modeling approach to guide the design of smaller C-terminal truncations of NSD3S shown in Figure 2A. Positive controls were first validated by the GST pulldown assay using glutathione-conjugated beads to isolate overexpressed GST-MYC and to identify NSD3S in the MYC complex. Indeed, full-length NSD3S and NSD3S fragment T-a (347–645) were found in complex with MYC (Figure 2B). Deletion of NSD3S residues 347–645 appeared to reduce, though not completely abolish, NSD3S interaction with MYC, suggesting a potential binding site within the region T-b (356–426). In support of this notion, NSD3S fragments lacking residues 356–426 exhibited no binding to MYC (Figure 2B). To test whether a MYC-binding site in NSD3S region 356–426 could be further delineated, we generated smaller truncations based on computational modeling predictions (Figure 2C). To guide the design of structural elements for testing, a computational molecular modeling approach was employed to define potential sub-structural regions within NSD3S fragment 356–426. The model obtained after a 100-ns molecular dynamics (MD) simulation suggested potential α-helical structures in regions W368–K377 and R383–Q389 of NSD3S, while the region encompassing Y390–L426 is largely disordered (Figure 2C). The secondary structures (SS) observed for NSD3S region 356–426 in this model agree with predictions made using Prime software (Jacobson et al., 2004). Based on these secondary structure predictions, refined truncations within residues 356–426 (T-b) of NSD3S were generated (Figure 2D). VF-NSD3S truncations were tested for interaction with GST-MYC by GST pulldown with glutathione beads (Figure 2D). Positive binding signals were detected between full-length MYC and NSD3S truncations T-g (378–404) and T-i (378–426) (Figure 2E). In contrast, no binding was observed between full-length MYC and NSD3S fragments T-f (356–378) and T-h (404–426), supporting a specific role for NSD3S residues 378–404 in mediating binding to MYC (Figure 2E). The 27-amino acid MYC-binding peptide of NSD3S was further divided into two segments (Figure 2F). Co-expression of GST-MYC and VF-NSD3S truncations was followed by the GST pulldown assay as described above. NSD3S residues 389–404 demonstrated interaction with MYC, while the peptide containing residues 378–389 failed to show positive interaction (Figure 2G). Our results indicate that the 15-amino acid peptide spanning residues 389–404 on NSD3S (NSD3S-pep15) is sufficient to enable interaction with MYC protein.

### NSD3S-pep15 is required for functional regulation of MYC

**Figure 3:**
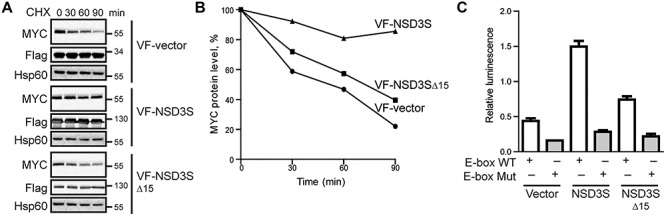
Deletion effect of NSD3S binding peptide on MYC transcriptional activity and stability. (**A**) Effect of NSD3SΔ15, on MYC stability by CHX treatment in HEK293T cells. Levels of endogenous MYC protein were determined at different time points after inhibition of protein synthesis by CHX with VF-vector, VF-NSD3S, or VF-NSD3SΔ15 overexpression. (**B**) Quantification of western blot showed in **A** of MYC levels at different time points of CHX treatment based on densitometric analysis. MYC protein levels were normalized to Hsp60 protein levels as the loading control. (**C**) Effect of NSD3SΔ15 on MYC reporter transcriptional assay in HEK293T cells. Luciferase activity was measured under conditions overexpressing VF-vector, VF-NSD3S, or VF-NSD3SΔ15 with either wild-type (WT) or mutant (Mut) E-box luciferase reporter plasmid. Relative luminescence is the ratio between luciferase and the internal Renilla activity control. Shown is a representative result of three independent experiments. The error bars show mean ± SD of three replicates.

To probe for a functional role of NSD3S residues 389–404 in the regulation of MYC, we generated a deletion mutant of NSD3S lacking residues 389–404 (NSD3SΔ15). It has previously been demonstrated that NSD3S overexpression can extend the half-life of MYC (Li et al., 2017); thus, the effect of NSD3SΔ15 deletion on MYC protein half-life was evaluated. Cell treatment with a protein synthesis inhibitor, cycloheximide (CHX), allows the monitoring of half-life of the MYC protein over time (Liu and Eisenman, 2012), which was used for the study. Consistent with our previous results (Li et al., 2017), overexpression of NSD3S stabilized MYC protein levels and increased MYC half-life. Compared to NSD3S, overexpression of NSD3SΔ15 exhibited a reduced stabilization effect on MYC, suggesting a partial role for this 15-residue NSD3S sequence in the stabilization of MYC (Figure 3A and B). Extended MYC protein half-life has been correlated with increased MYC transcriptional activity. To test the effect of NSD3SΔ15 on MYC-driven transcription, an E-box-dependent luciferase-based MYC reporter assay was used (Yin et al., 2003; Jung et al., 2015). Consistent with our previous report, overexpression of NSD3S was correlated with increased MYC transcriptional activity as shown by increased E-box-dependent luciferase signal (Li et al., 2017; Figure 3C). In contrast, expression of NSD3SΔ15 failed to fully activate the MYC transcriptional activity, showing significantly decreased luciferase signal compared to that with NSD3S (Figure 3C). These data demonstrate that NSD3S residues 389–404 are crucial for NSD3S enhancement of MYC transcriptional activity. Together, these results suggest that amino acids 389–404 of NSD3S are partially required to regulate MYC function, increasing protein half-life and transcriptional activity.

### NSD3S stabilizes MYC by interfering with FBXW7-mediated proteasomal degradation

**Figure 4:**
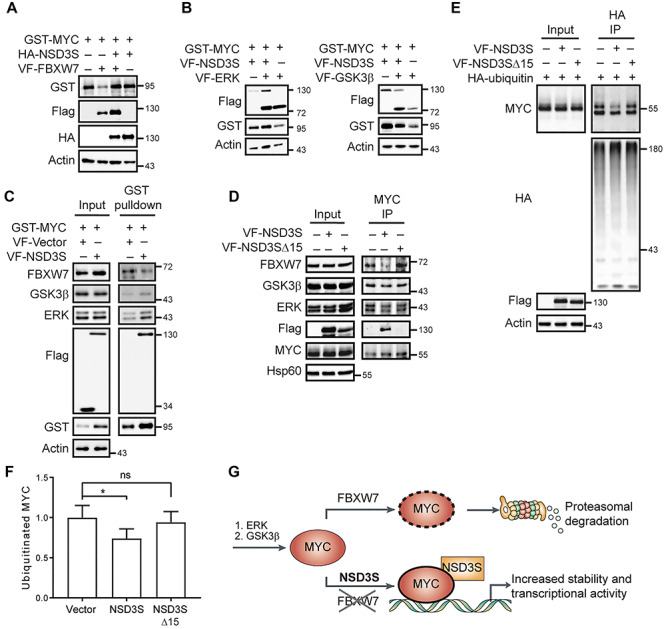
Effect of NSD3S on MYC proteasomal degradation by FBXW7. (**A**) HEK293T cell lysate for the detection of GST-MYC levels with NSD3S in combination with FBXW7. Levels of MYC were compared against NSD3S or FBXW7 alone, and actin was used as a loading control. (**B**) Detection of MYC levels on cell lysate from HEK293T with NSD3S in combination with GSK3β or ERK. MYC levels were compared against NSD3S, GSK3β, or ERK alone, and actin was used as a loading control. (**C**) Detection of endogenous binding of FBXW7, GSK3β, and ERK to GST-MYC in the presence of NSD3S by GST pulldown. Protein levels detected in the pulldown were compared to that in the input, and to actin as a loading control in HEK293T cells. (**D**) Evaluation of FBXW7 binding to MYC by co-IP in the presence of NSD3S constructs. Endogenous FBXW7 in complex with endogenous MYC was immunoprecipitated with MYC antibody. Protein levels were compared to that in the input, and Hsp60 protein was used as a loading control in HEK293T cells. (**E**) Determination of ubiquitinated MYC levels by HA-immunoprecipitation (HA-IP) of HA-ubiquitin in the presence or absence of NSD3S or NSD3SΔ15. Levels of endogenous MYC detected in the HA-ubiquitin complex were compared to that in the input and to actin as a loading control in HEK293T cells. (**F**) Quantification of western blot shown in **E** of ubiquitinated MYC levels based on densitometric analysis. MYC ubiquitination levels are presented as means ± SD normalized to vector control levels; *n* = 4. One-way ANOVA with Dunnett’s test for multiple comparisons was performed to compare conditions; *P = 0.0415; ns, *P* = 0.7809. (**G**) Proposed model for NSD3S role in MYC protein stability. NSD3S impacts a major pathway, the FBXW7-mediated proteasomal degradation pathway, which regulates MYC half-life. NSD3S binds to MYC and suppresses the FBXW7 activity by disrupting the interaction with MYC, increasing MYC protein half-life and transcriptional activity.

MYC is a transcription factor that acts as a master regulator of gene expression in cancer. Because of its significant role in regulating different characteristics of cancer cells to promote tumorigenesis, MYC activity is tightly regulated through intracellular PPIs (Tu et al., 2015). To further understand the mechanism of MYC stabilization by NSD3S, we investigated potential effects of NSD3S on MYC binding partners known to regulate MYC stability and degradation. One of the most extensively characterized proteolytic degradation pathways for MYC involves FBXW7, a substrate recognition subunit of SCF E3 ubiquitin ligase complexes. For FBXW7 to associate with and mediate ubiquitination of MYC, MYC requires phosphorylation by two kinases, ERK and GSK3β (Thomas and Tansey, 2011). Interestingly, expression of FBXW7 reduced MYC protein levels as expected, while co-expression of NSD3S with FBXW7 increased MYC levels, suggesting an antagonistic effect of NSD3S on FBXW7 function (Figure 4A). Because ERK and GSK3β are well established regulators of MYC, the effect of NSD3S on MYC stability was examined under conditions with differential expression of ERK or GSK3β. As shown in Figure 4B, co-expression of NSD3S with either ERK or GSK3β exhibited MYC levels that were comparable to those under conditions of ERK, GSK3β or NSD3S overexpression alone.

Since NSD3S increases MYC protein stability and suppresses the action of FBXW7 on degrading MYC, we tested whether NSD3S expression could influence the interaction between FBXW7 and MYC. We performed a GST pulldown assay against GST-MYC protein with overexpression of VF-NSD3S, or VF-vector control. Overexpression of VF-NSD3S correlated with reduced interaction of endogenous FBXW7 with GST-MYC in HEK293T cells (Figure 4C). As in Figure 4B, NSD3S overexpression resulted in no changes in the interactions between MYC/ERK or MYC/GSK3β (Figure 4C). We then evaluated whether NSD3S was able to disrupt endogenous binding between MYC and FBXW7. We performed an endogenous co-immunoprecipitation (co-IP) in HEK293T cells using a MYC antibody, with overexpressed VF-vector, VF-NSD3S, or VF-NSD3SΔ15. In agreement with the results in the MYC overexpression system, NSD3S overexpression resulted in reduced interaction between endogenous FBXW7 and MYC but had no effect on the association of ERK or GSK3β with MYC (Figure 4D). In contrast, NSD3SΔ15 overexpression did not inhibit the interaction between endogenous FBXW7 and MYC (Figure 4D).

FBXW7 is an SCF E3 ubiquitin ligase subunit that targets proteins for ubiquitination and proteasomal degradation (Yeh et al., 2018). We thus investigated the effects of NSD3S expression on FBXW7-mediated ubiquitination of MYC. Hemagglutinin (HA)-tagged ubiquitin was overexpressed and immunoprecipitated using anti-HA antibodies, and ubiquitination of endogenous MYC was evaluated. NSD3S overexpression resulted in a decrease in MYC ubiquitination, while NSD3SΔ15 overexpression resulted in a level of MYC ubiquitination similar to vector control (Figure 4E and F). Disruption of the interaction between MYC and FBXW7 by NSD3S thus correlates with decreased MYC ubiquitination and increased MYC protein levels in cells (Figure 4G). Taken together, these results suggest a novel mechanism for how NSD3S stabilizes MYC protein levels by opposing the FBXW7-mediated degradation pathway.

## Discussion

MYC is a master regulator of cell growth and its activity is tightly regulated by diverse mechanisms (Chakravorty et al., 2017). Here we present evidence in support of a novel mechanism by which NSD3S stabilizes MYC through disruption of the FBXW7/MYC interaction.

To gain insights into the molecular interaction between NSD3S and MYC, we identified an internal region of MYC that extends through MBIII and MBIV as a NSD3S binding site (Figure 1D). In contrast to the MBI and MBII regions that are localized in the transactivation domain and are important for cell transformation (Kato et al., 1990), and the C-terminal bHLH-LZ domain that is important for MAX dimerization and DNA binding, the internal segment of MYC is highly disordered and the functions of MBIII and MBIV have not been extensively characterized. In addition, most of the MYC binding partners bind to the N- or C-terminus of the protein (Blackwood and Eisenman, 1991; Tu et al., 2015; Kalkat et al., 2018). For example, these binding sites include ERK (N-terminal) (Lutterbach and Hann, 1994; Sears et al., 2000), GSK3β (residues 1–100) (Lutterbach and Hann, 1994; Gregory et al., 2003), and FBXW7 (MBI, residue phospho-T58) (Welcker et al., 2004; Yada et al., 2004). Thus, mapping the binding site for NSD3S reveals a new functional site on MYC with a potential regulatory role for MYC stabilization.

On NSD3S, we narrowed down the MYC binding site to a 15-amino acid peptide. NSD3S has one defined structural domain, termed the PWWP domain, which has been shown to mediate binding to methylated H3K36me2/3 (Wu et al., 2011) and other proteins (Stec et al., 2000). Currently, only two NSD3 binding proteins have been mapped: BRD4, which binds to amino acids 152–163, and CHD8, which binds to C-terminal amino acids 384–645 (Shen et al., 2015). Here, we add MYC as a binding partner of NSD3S that specifically binds to residues 389–404. Importantly, our data reveal a critical role for NSD3S-pep15 in the regulation of MYC function, as deletion of this segment leads to a partial decrease in MYC transcriptional activity and degradation.

MYC is an ‘immediate early gene’ (Marcu, 1987; Spencer and Groudine, 1991) known to be a serum-activated protein with a short half-life of ∼30 min in proliferating cells (Hann and Eisenman, 1984). Different regulatory mechanisms have been reported to alter MYC protein stability that could directly impact MYC signaling under physiological and pathological conditions (Farrell and Sears, 2014). One well-established mechanism is the priming of MYC by two kinases, ERK and GSK3β, which leads to the binding of FBXW7, a protein that directly regulates MYC degradation (Adhikary and Eilers, 2005). Previously, we reported that NSD3S increases MYC protein stability and that the stabilization effect is possibly through binding (Li et al., 2017). Our current findings reveal that NSD3S stabilizes MYC by interfering with the interaction between MYC and FBXW7, an E3 ligase subunit known to mediate MYC ubiquitination and degradation, in part through a 15-amino acid region of NSD3S (Welcker et al., 2004, Yada et al., 2004). NSD3S does not influence ERK or GSK3β activity, but it appears to suppress FBXW7 function (Figure 4F). Interestingly, NSD3S interferes with FBXW7 binding to MYC, preventing ubiquitination and proteasomal degradation of MYC. GSK3β phosphorylation of MYC at T58 allows interaction of FBXW7 with MYC through its MBI domain (Salghetti et al., 2000). Here we show that NSD3S binds to the internal region of MYC (residues 186–338). Our data reveal that NSD3S and FBXW7 bind to different regions of MYC. It remains to be determined how exactly NSD3S interferes with FBXW7 binding to MYC. However, it is possible that the binding of NSD3S to MYC alters the three-dimensional conformation of MYC such that FBXW7 binding to MYC is hindered. Another possibility is that NSD3S affects posttranslational modification events on MYC that are required for FBXW7 binding (Wang et al., 2018), such as the phosphorylation and dephosphorylation of MYC on residues T58 and S62 or the binding of peptidyl-prolyl cis-trans isomerase NIMA-interacting 1 (Pin1) or protein phosphatase 2 (PP2A) to MYC. Further studies will be required to elucidate this mechanism.

NSD3S belongs to the NSD family of proteins that have been implicated in oncogenesis (Morishita and di Luccio, 2011). Specifically, NSD3S, a short isoform of the NSD3 histone lysine methyltransferase, lacking methyltransferase activity, has been linked with progression of leukemia (Shen et al., 2015) and NUT-midline carcinoma (French et al., 2014; Suzuki et al., 2014). Here, we report a novel mechanism for how NSD3S may contribute to tumorigenesis by acting as a positive regulator of the MYC oncogene. Moreover, this regulation shows a broader mechanism of action of NSD3S, as MYC acts as a driver in multiple solid and hematological cancers (Vita and Henriksson, 2006) and this study suggests a regulation of MYC by NSD3S in various cellular environments.

Results from this paper defined a binding interface between NSD3S and MYC, which may provide a basis for further understanding of the interaction for functional manipulation. This study also revealed the importance of NSD3 residues 389–404 in MYC regulation, which may lead to future strategies to regulate MYC function. Furthermore, we uncovered a novel regulatory mechanism of MYC by NSD3S binding, adding a novel regulatory element to the well characterized FBXW7 proteasomal degradation pathway for MYC. This regulatory circuit between FBXW7/MYC/NSD3S makes the MYC/NSD3S PPI an interesting target for therapeutic exploration in MYC-driven tumors, as it impacts both transcriptional activity and the half-life of MYC protein. Targeting the MYC/NSD3S PPI interface may prove beneficial for patients with MYC amplification, as their disruption may restore the binding between MYC and the tumor suppressor FBXW7.

## Materials and methods

### Plasmids and reagents

Plasmids for mammalian expression of GST-, VF-, and human influenza HA-tagged proteins were generated using the Gateway cloning system (Invitrogen) according to the manufacturer’s protocol. Human MYC and NSD3S cDNA were used as previously described (Li et al., 2017). FBXW7 and ERK cDNA were obtained from DNASU, and GSK3β was provided by Dr Kenneth Scott. MYC and NSD3S truncations were generated by introducing a stop codon using a PCR-based mutagenesis protocol. All plasmids were confirmed by FastDigest Bsp1407I (ThermoScientific, Cat# FD0934) enzyme digestion and by sequencing analysis. The protein synthesis inhibitor, CHX (Cell Signaling Technology, Cat# 2112), was used for the MYC stability assays at a final concentration of 100 μg/ml. The proteasome inhibitor, MG-132 (Cell Signaling Technology, Cat# 2194), was used at a final concentration of 20 μM for 3 h before cell collection.

### Cell culture

HEK293T embryonic kidney cells (ATCC CRL-3216) were obtained from American Type Cell Culture Collection (ATCC) and maintained in Dulbecco’s modified Eagle’s medium (VWR, Cat# 45000-304). Culture media was supplemented with 10% fetal bovine serum and 1% penicillin/streptomycin solution (CellGro, Cat# 30-002-CI). Cells were cultured at 37°C with 5% CO_2_ in a humidified incubator. Between passages, cells were detached with 0.25% trypsin with EDTA (VWR, Cat# 45000-664).

### Gene expression

HEK293T was transfected with expression plasmids at a confluence of 60%–70% using X-tremeGENE (Roche, Cat# 06366546001) transfection reagent. A ratio of 3 μl transfection reagent to 1 μg of plasmid DNA was utilized in a volume of 100 μl of Opti-MEM reduced serum medium (Thermo Fisher, Cat# 31985070) for plasmid delivery, according to the manufacturer’s instructions. Expression of transfected genes was monitored by western blotting with corresponding antibodies.

### GST pulldown and immunoprecipitation

HEK293T cells were seeded in 6-well plates and transfected. After 48 h, cells were collected and lysed with 200 μl of lysis buffer (150 mM NaCl, 10 mM HEPES, pH 7.5, 0.25% Triton X-100, phosphatase inhibitor (Sigma, Cat# P5726) and protease inhibitor cocktail (Sigma, Cat# P8340)) by sonication. Lysate was centrifuged to eliminate cellular debris, among which, 20 μl was saved for an input control, and the rest was incubated with 20 μl of 50% glutathione-conjugated sepharose beads (GE, Cat# 17-0756-05) for GST pulldown experiments, 5 μl of EZview Red Anti-Flag M2 Affinity Gel (Sigma, Cat# F2426) for Flag-IP, or 10 μl of EZview Red Anti-HA Affinity Gel (Sigma, Cat# E6779) for HA-IP for 3 h rotating at 4°C (Rusnak et al., 2018). Beads were washed three times in 0.25% Triton X-100 lysis buffer and eluted by boiling for 5 min in sodium dodecyl sulfate (SDS) loading buffer. Samples were then analyzed by SDS–polyacrylamide gel electrophoresis (SDS–PAGE) and western blotting.

### MYC reporter assay

HEK293T cells were seeded in 12-well plates. The next day, cells were transfected with VF-plasmids along with firefly luciferase reporter plasmid under the control of wild-type (GCCACGTGGCCACGTGGCCACGTGGC) or mutant (GCCTCGAGGCCTCGAGGCCTCGAGGC) E-box and Renilla luciferase, which served as an internal control. Cells were collected, centrifuged at 1200 rpm, and re-suspended in 50 μl of phosphate-buffered saline (VWR, Cat# 45000-446). 10 μl of cells was transferred to a 384-well plate, and the MYC reporter assay was performed using Dual-Glo luciferase kit (Promega, Cat# E2920) following the manufacturer’s instructions. Firefly luciferase expression was normalized to the control Renilla expression. Data were analyzed on Graphpad Prism software.

### Protein stability assay

HEK293T cells were seeded on 24-well plates and transfected. After 48 h, cells were treated with 100 μg/ml of CHX. At the indicated times, cells were collected with 80 μl of 2× SDS loading buffer, boiled for 10 min, and resolved by SDS–PAGE. Protein expression was detected by western blotting and quantified using GelQuant software. Hsp60 protein levels were used as an internal control for MYC protein normalization.

### Endogenous co-IP

HEK293T cells were plated on 15 cm dishes and transfected. After 48 h, cells were collected and lysed with 0.25% Triton X-100 lysis buffer by sonication. Lysate was centrifuged and 40 μl of the supernatant was saved for an input control; the rest was incubated with MYC antibody (Santa Cruz, Cat# sc-40) overnight at 4°C. Then, protein A/G PLUS-agarose beads (Santa Cruz, Cat# sc-2003) were added to the mixture and were incubated at 4°C for another 4 h. Beads were washed three times with 0.25% Triton X-100 lysis buffer, and proteins were eluted with SDS–PAGE loading buffer and analyzed by western blotting.

### Antibodies

The following antibodies and conjugates were used for the western blotting: Flag-HRP (Sigma, Cat# A8592, dilution 1:2000), GST-HRP (Sigma, Cat# A7340, dilution 1:3000), HA-HRP (Sigma, Cat# H6533, dilution 1:3000), goat anti-mouse IgG-HRP (Jackson ImmunoResearch, Cat# 115-035-003, dilution 1:3000), and goat anti-rabbit IgG-HRP (Jackson ImmunoResearch, Cat# 111-035-003, dilution 1:3000), and antibodies for MYC (Cell Signaling Technology, Cat# 5605S, dilution 1:1000), MYC (Santa Cruz, Cat# sc-40, dilution 1:1000), FBXW7 (Abcam, Cat# ab109617, dilution 1:1000), ERK (Cell Signaling Technology, Cat# 4695, dilution 1:1000), GSK3β (Cell Signaling Technology, Cat# 9315, dilution 1:1000), actin (Sigma, Cat# A5441, dilution 1:3000), GFP (Abcam, Cat# ab290, 1:6000), and Hsp60 (Cell Signaling Technology, Cat# 4870, dilution 1:1000).

### Computational modeling

The model of NSD3S region 356–426 was built using the Schrodinger Prime software. The BLAST search was utilized to identify the template proteins with highest sequence similarity. The SS analysis of NSD3S 356–426 was performed with the Prime program and matched with the SS of the template proteins. The BLAST search of the crystallized proteins available in the Protein Data Bank has revealed that NSD3S fragment 356–396 demonstrates the highest sequence and structure similarity (prime alignment score: 89, sequence similarity: 63%) with the crystallized region of PWWP1 domain of WHSC1 protein (PDB ID: 5VC8). Therefore, this structure was utilized as a template to construct NSD3 fragment 356–396. Since further residues of WHSC1 were not solved in the crystal structure, we sought for another template protein to build a model of NSD3 397–426. Through the BLAST and the SS analysis, the structure of Parkin E3 ligase (PDB ID: 4I1H) was identified as the most suitable temple (prime alignment score: 55.0; sequence similarity: 60%). Since the crystal structures of WHSC1 and the Parkin E3 ligases lack similar regions for structural alignment, NSD3 356–396 and NSD3 356–426 were first built separately, and then connected using the Schrodinger Crosslink Proteins tool. The resulting structure of NSD3 356–426 was optimized by energy minimization in the OPLS2005 force field using the Polak-Ribier Conjugate Gradient algorithm. First, only the protein side chains were subjected to the energy minimization by 2500 interactions and the convergence threshold of 0.05. Then, the constraints were released, and the whole structure of NSD3 356–426 was optimized by 10000 interactions and the convergence threshold of 0.05.

The resulting model was further optimized by 100-ns MD simulation performed with the Desmond program implemented in the Schrodinger Suite. The NSD3S model was placed in a box that contained 9852 SPC water molecules and 8 chloride anions. The resulting system contained a total of 30791 atoms. The MD simulation was run for 100 ns under the normal temperature and pressure conditions, with the following parameters: Thermostat method: Nose-Hoover chain, relaxation time 1.0 ps; Barostat method: Martyna-Tobias-Klein, relaxation time 2.0 ps, time step 2 fs, short range cutoff radius 9.0 Å, energy recording time 1.2 ps, trajectory recording time 10 ps. The energy of the resulting structure of NSD3S 356–426 obtained after the MD simulation was minimized as described above.

### Data analysis

All data were performed and repeated three times. The data quantification of the MYC reporter and protein stability assays was performed using the GraphPad Prism software.
